# The experiences of students with mental health difficulties at medical school: a qualitative interview study

**DOI:** 10.1080/10872981.2024.2366557

**Published:** 2024-06-13

**Authors:** Antonia Rich, Rowena Viney, Milou Silkens, Ann Griffin, Asta Medisauskaite

**Affiliations:** a Research Department of Medical Education, UCL Medical School, University College London, London, UK; b Centre for Healthcare Innovation Research, Department of Health Services Research and Management, School of Health and Psychological Sciences, City University of London, London, UK; c Erasmus School of Health Policy and Management, Erasmus University Rotterdam, Rotterdam, the Netherlands

**Keywords:** Undergraduate medical education, qualitative study, mental health, help-seeking, medical culture

## Abstract

**Background:**

High rates of burnout, anxiety, and depression in medical students are widespread, yet we have limited knowledge of the medical school experiences of students with mental health issues. The aim of the study is to understand the impact of mental health issues on students’ experience and training at medical school by adopting a qualitative approach.

**Methods:**

Qualitative study using in-depth semi-structured interviews with 20 students with mental health issues from eight UK medical schools of varying size and location. Students were purposefully sampled to gain variety in the type of mental health issue experienced and demographic characteristics. Reflexive thematic analysis was employed using NVivo software.

**Results:**

Three themes were identified. 1) Culture of medicine: medical culture contributed to causing mental ill-health through study demands, competitiveness with peers, a ‘suck it up’ mentality where the expectation is that medical school is tough and medical students must push through, and stigma towards mental ill-health. 2) Help-seeking: students feared others discovering their difficulties and thus initially tried to cope alone, hiding symptoms until they were severe. There were multiple barriers to help-seeking including stigma and fear of damage to their career. 3) Impact on academic life: mental health issues had a detrimental impact on academic commitments, with students’ unable to keep up with their studies and some needing to take time out from medical school.

**Conclusion:**

This study provides insight into how medical culture contributes both to the cause of mental health difficulties and the reluctance of medical students to seek help. Mental health issues had a considerable negative impact on medical students’ ability to learn and progress through their degree. Addressing the medical culture factors that contribute to the cause of mental health issues and the barriers to help-seeking must be a priority to ensure a healthier medical workforce.

## Background

Increasingly research has drawn attention to the rates of mental ill-health within the medical student population, with large-scale meta-analyses noting a high prevalence of burnout, anxiety, and depression [1–3]. Approximately one in three medical students suffer from depression, depressive symptoms or anxiety [2,3] and rates of depression and anxiety in medical students have been found to be higher than their non-medical student peers [4]. Whilst there is less data available about the prevalence of death by suicide in medical students compared to the wider non-medical student population, one in ten report suicidal ideation [3]. These figures give an indication of the severity of the situation and concern has grown in recent years as the prevalence of mental illness amongst medical students has become more prominent.

The causes for such distress include academic and financial pressures, competition with peers, the clinical environment with exposure to death, suffering and ethical conflicts [5]. Further, stigma towards mental health and concerns for the implications for future career prospects creates a population that is reluctant to disclose difficulties and seek help [6,7]. While research has helped us understand the factors that contribute toward mental health in the general population of medical students, much less is known about the experiences of medical students with mental health issues. In this study, we aim to understand the factors specific to medical school that may have contributed to mental health issues and the impact of mental health on students’ experience and training at medical school. Such understanding is necessary to help identify the needs of medical students with mental health issues, so these students can be supported during their studies. To our knowledge this is an original study utilising in-depth interviews to investigate the experiences of medical students with mental health issues from eight UK medical schools.

## Methods

### Design

The research was part of a larger research study consisting of a questionnaire, interviews, and documentary analysis investigating medical students’ mental health. Eight UK Medical Schools participated in the interview arm of the study and varied in size, location across the UK and type of curriculum (integrated/non-integrated). In the UK, standard entry medical students currently undertake up to six-years of undergraduate study. Typically, early years curriculum covers the basic medical sciences and later years shifts to cover clinical education and workplace-based learning. The emphasis on placement learning, early patient contact and teaching methods varies according to the school.

This qualitative study involved in-depth semi-structed interviews which took place via Microsoft Teams in 2021. Questions were deliberately open-ended to enable the interviewees to discuss their own experiences of mental health, without being unduly guided by the interviewer, and for the interviewer to follow-up areas of particular interest. The interview guide can be found in the supplementary material. The interviews were video recorded, and the audio was professionally transcribed ‘intelligent verbatim’ (capturing all talk except speech disfluencies such as ‘um’ and ‘er’ and sentence re-starts). Participants were given a certificate for taking part and a list of support services for mental ill-health tailored to their university and geographical location. A COREQ checklist is provided as a supplementary file.

## Participants and recruitment

Students completed a survey about their mental health where they were able to express an interest in taking part in a subsequent interview. To be eligible to participate, students had to have experience of a mental health issue whilst at medical school. They were advised against participating if they were acutely unwell. To be eligible to participate it was not a requirement to have a clinical diagnosis. A total of 130 students volunteered to participate and were sent a short follow-up questionnaire to obtain their demographic details. Students were presented with a list of possible mental health issues. They were asked to indicate the type of mental health issue they had experienced, if they felt comfortable to do so, from the following options: depression, anxiety, insomnia, stress, burnout, eating disorder, drug or alcohol use, personality disorder, bipolar disorder and other (please state). Students were then purposefully sampled to acquire diversity, taking into consideration the following: type of mental health issue, medical school, gender, year of study, ethnicity, and sexuality (See Table 1). Selected students were emailed an invitation to participate which provided a comprehensive information sheet. Participants were asked to complete an online consent form via the secure online data collection tool REDCap which captures data directly into UCL’s Data Safe Haven, appropriate for special category personal data. Ethical approval was obtained by UCL Ethics Committee (REF: 14983/002) and data protection registration with UCL’s data protection office (REF: No Z6364106/2019/11/32) in line with the General Data Protection Regulation 2018.

**Table 1.** Interview participants.

Demographic Category	Participant Numbers (%)
Gender	
Female	12 (60%)
Male	7 (35%)
Non-binary	1 (5%)
Year of Study	
Early (years 1–3)	9 (45%)
Late (years 4–6)	11 (55%)
Ethnicity	
Black	1 (5%)
Arab	1 (5%)
Asian	7 (35%)
Mixed	2 (10%)
White	9 (45%)
Sexuality	
Asexual	1 (5%)
Bisexual	4 (20%)
Gay/lesbian	1 (5%)
Heterosexual	13 (65%)
Other	1 (5%)
Mental Health Issue*	
Anxiety	12 (60%)
Bipolar	1 (5%)
Burnout	12 (60%)
Depression	14 (70%)
Drugs and alcohol misuse	3 (15%)
Eating disorders	6 (30%)
Insomnia	6 (30%)
Personality disorder	2 (10%)
Stress	14 (70%)

*Students reported experiencing multiple mental health conditions or symptoms, thus the total numbers for condition do not add up to 20. Each medical school had between one to three participants.

### Patient and public involvement

A Steering Group met throughout the study which comprised two medical student representatives, in addition to members from Medical Schools Council, Practitioner Health Programme, British Medical Association and academics with experience of researching medical student mental health. The Steering Group provided feedback and advice on all aspects of the study including recruitment, development of interview questions, data analysis, and dissemination of the research.

## Analysis

Three researchers (AR, RV, MS) conducted the interviews and undertook reflexive thematic analysis [8,9]. Reflexive thematic analysis is a theoretically flexible interpretative approach to qualitative data analysis that enables the systematic identification, organisation and analysis into themes within a data set [10,11]. Reflexive thematic analysis was considered an appropriate method for the current study due to our wish to undertake an inductive analysis which would enable the identification of themes in a sample with diverse characteristics as outlined in Table 1 [10,12]. In addition, reflexive thematic analysis was chosen because of its aim to identify how personal experiences are located in the wider social-cultural context and focus on producing results with implications for practice [10,12,13]. The researchers have differing professional backgrounds (AR-health psychologist, RV-linguist and MS-medical educationalist) and considerable experience conducting qualitative research. The researchers familiarised themselves with the transcripts and checked against the original audio recordings for accuracy. Initial codes were generated by each researcher for a randomly selected transcript and discussed as a team. This was repeated for two subsequent transcripts. This allowed the researchers to develop a coding framework, which was refined as the iterative analysis process progressed. The remaining transcripts were divided among the three researchers for coding, who met throughout the analysis process to discuss coding and patterns in the data, which enabled development of themes and sub-themes. Detailed development of the coding framework and analysis has been published elsewhere [12]. To allow the reader to judge the trustworthiness of the study, we demonstrate how the study attempts to meet the criteria outlined by Lincoln and Guba [14] of credibility, dependability, confirmability, and transferability (please see discussion).

## Results

Interview duration ranged from 35 to 61 minutes (mean = 50 minutes). The findings from the Reflexive thematic analysis have been organised into three primary themes, each with a number of sub-themes (Table 2). We present the findings from the qualitative interviews supported by quotations which describe participants’ experiences in their own words, and how they make sense of their mental health whilst at medical school.

**Table 2.** Themes and sub-themes resulting from Reflexive Thematic Analysis.

Theme	Sub-themes
(1) Culture of medicine	Study demands Competitiveness “Suck it up” culture Stigma
(2) Help-seeking	Hiding symptoms Symptom severity and delays to treatment Fear of damage to career
(3) Impact on academic life	Effect on studies Time out from medical school

### Theme 1. Culture of medicine

#### Study demands

Students described feeling overwhelmed due to the intensity of work demands from medical school, particularly the quantity of their workload, with a high number of lectures and volume of information per lecture. Juggling a busy academic timetable with exams and clinical placements could feel relentless, creating a huge amount of pressure for students struggling to keep on top of their degree’s requirements. Medical schools did often promote the message of self-care, such as taking time for yourself, however students often did not feel they had the time to employ the strategies of self-care suggested and thus this message was received with scepticism:

*Ultimately if you’re putting people under that level of stress you know say “take time for yourself” is … you know take time for yourself, make sure you eat … blah blah blah … it’s like well fantastic if I wasn’t working for 15 hours a day trying to get through these ridiculous number of lectures*. (*Participant 1)*

Mental ill-health symptoms typically fluctuated throughout the year but would often be triggered by study-related stress, peaking with the pressure of exams:

*I have very distinct memories of second year exams where I would just sit on the couch and my flatmates would just like sit near me and make sure I was okay because I just physically couldn’t move. I’d go from like shaking constantly for hours and then I would just stop moving completely just from like the stress that I was feeling. (Participant 19)*

For some this was related to pressure from the desire to achieve high grades. For others, the pressure was due to a fear of failure. Disappointment in grades could create feelings of despondency, negatively affecting their confidence, and doubts about their choice of a medical career. Failing an exam is high stakes as it could result in permanent repercussions such as expulsion from their degree:

*That feeling of if I don’t pass these exams then I’m out and all this that I’ve worked towards you know I won’t have achieved what I was aiming to achieve, so it’s just that fear of failure I suppose. (Participant 13)*

#### Competitiveness

Participants described a competitive culture, with peers constantly comparing themselves with one another and feeling that others appeared to cope with the workload better than them. Students were reluctant to voice their concerns not only to medical school staff, but also their social circle. This can create an isolating experience for those who are struggling. At its extreme, the intense competitive environment of medical school itself was described as *‘pathogenic’ (Participant 1)*, particularly at the transition to entry into medical school, because medical students by their nature are high achievers and, prior to medical school, are used doing well in comparison to their peer group.

Several felt their medical school exacerbated competitiveness. One student described how their school increased competitiveness by publishing students’ grades alongside one another. However, some students reported that their medical school tried to create a non-competitive environment, which was warmly welcomed. There were examples where their medical school tried to avoid comparison and be supportive, even normalising exam failure:

*the professors were very understanding and just saying that most people will fail in second year. Yeah. (laughs) Yeah so it was obviously a good support. (Participant 13)*

In addition to the requirements of their degree, students are strongly encouraged to do extra-curricular activities to ensure a competitive CV. It was acknowledged that medical students are *‘over-achievers’ (Participant 12)* and could feel compelled to take on additional activities, despite being already overwhelmed with their workload. Comparison with other high achievers created immense pressure and could lead to self-doubt, feelings of inadequacy and a loss of confidence with some questioning their ability to qualify as a doctor. Several participants reported experiencing imposter syndrome:

*And also I guess imposter syndrome also kicked in a lot because everyone around me felt so much smarter and better than me at everything, and I felt really out of place. (Participant 14)*

The pressure led to high levels of stress which could then trigger or exacerbate mental health issues. This can create a vicious circle in terms of negatively affecting a student’s ability to learn and manage their workload which in turn can worsen their mental health:

*when you’re not feeling mentally as good you can’t kind of keep up with the work and then you start worrying like I’m going to fall behind, everyone else is doing better than me – which just kind of adds into the cycle of not feeling good. (Participant 15)*

#### ‘Suck it up’ culture

Most students described their medical school as a high pressure, demanding and uncaring environment. The culture of medicine is one where students are required to be tough and must ‘suck up’ the challenges of medical school, without complaint:

*I think the expectation is that you know doctors are tough, you just sort of push forward and carry on. Medical school is tough you know it’s tough so just suck it up and get on with it. (Participant 6)*

Some students felt that even though the pressure and negative toll on their mental health was considerable, they were part of a culture where their emotional difficulties were considered inconsequential:

*like it doesn’t matter if you end up exhausted and crying all the time and all of this, like as long as you get through it. (Participant 8)*

It was felt by some that it was older doctors who tended to hold the ‘tough it out’ attitude. Older doctors made comments such as *‘we worked 100 hours a week’* and ‘*burnout didn’t exist when I was younger’ (Participant 7)*, whereas younger doctors were perceived as more understanding of the pressures and willing to help, thus the culture may be moving in a more positive, empathic direction.

#### Stigma

Stigma towards mental health was apparent in many aspects of medical culture, including at medical school and the NHS (The National Health Service (NHS) is the Government-funded medical and health care services that everyone living in the UK can access.

##### Stigma towards mental health at medical school

The implicit message at medical school was that being a medical student is incompatible with having a mental health issue. Mental health was felt to be deprioritised compared to other subjects in the curriculum: ‘*it’s kind of pushed to the side’ (Participant 20)*. During teaching sessions, stigmatisation of mental ill-health was apparent in descriptions of patients which were felt to be overly simplistic, drawing heavily on stereotypes *(Participant 11)*. People with mental illness diagnoses were also ‘othered’ during teaching; case studies were presented as something that happens to other people, not medical students:

*We had a mental health case last year, so we studied depression, anxiety, psychosis. One thing that I didn’t find particularly helpful during that case was how it was presented as something which we don’t necessarily experience ourselves … So I suppose during that case the presentation of it as something that somebody else experiences rather than something that we all have a degree of understanding of. (Participant 6)*

Some peers at medical school were also reported to perpetuate stigma around mental health. When discussing case studies, peers were described as using ‘*language which is either derogatory or insensitive’ (Participant 3)*, and displaying negative stereotypes of mental health patients, for example ‘*this person is depressed because they’re unclean and they haven’t got a job’ (Participant 8)*. Stigma was also conveyed in general conversations with peers, with several students reporting hearing insulting comments from others about people with mental health conditions. This led to students with mental health issues feeling alone, as they did not feel accepted as part of medical culture:

*On the first day I met somebody who was like “Oh I can’t be a GP, because if somebody comes in with depression I’ll tell them to get over themselves” – like there are many people that think that way. Which I think is not like doctor material – but that’s another discussion. So I did feel just a little bit alone. (Participant 8)*

However, there was some variability in experiences, with not all students feeling that mental health was stigmatised by their peers:

*I think most of the other students are quite like forward thinking about it, like oh yes mental health is real, it’s valid, it’s something that you need to seek support for*. *(Participant 20)*

##### Stigma within the NHS

Medical students described incidences of stigma directly from doctors and other health professionals whilst accessing the NHS for treatment as patients themselves. One student was told by their GP that it was impossible to be a doctor with a mental health issue and questioned their career choice:

*And then when I told him [GP] I was a medical student he said “Oh this isn’t something that medical students should really be dealing with if you want to be a doctor, this shouldn’t be happening … maybe you should think about what you’re doing as well”. (Participant 2)*

Similarly, a student who was admitted to A&E (The Accident and Emergency (A&E) department at UK hospitals is for major, life-threatening illnesses and injuries),due to self-harm received judgemental comments from staff, such as *‘Oh you should know better’ (Participant 11).*

### Theme 2. Help-seeking

#### Hiding symptoms

Students feared their mental health issue being discovered, wanting to appear able to cope with the demands of medical school. This led to some deliberately concealing their condition to ‘*keep everything hidden’ (Participant 11)*, causing additional stress. Students would modify their behaviour to reduce the likelihood that others would notice them. For example, a student who feared having a panic attack in a lecture theatre would sit in a specific place so that she would not attract the attention of others if she needed to leave. Students could feel self-conscious about taking time off from their studies to attend treatment sessions and would conceal the true reason for their absence from friends. This contributed to a sense of isolation at medical school because students were reluctant to disclose to medical school friends about their situation, choosing instead to confide in non-medical friends.

#### Symptom severity and delays to treatment

Several of the students experienced symptoms over a long period which had gone untreated and worsened in severity over time:

*But the signs were there that things weren’t right, because for example I think I self-harmed twice in first year, but then exponentially increased by second year. (Participant 9)*

Symptoms were often severe before help was sought. Failing an exam, advice or assistance from a friend were given as examples of turning points for students to eventually seek help:

*I couldn’t even leave the flat without having a panic attack and that’s when … like basically my flatmate literally physically walked me to the GP over the road. (Participant 11)*

Students were reluctant to seek help both from their medical school and from their GP because of perceived stigma attached to being a medical student or doctor with a mental health condition. There was also the fear of meeting another health professional they knew in a professional capacity and being judged. One student gave an example of taking an overdose and despite being heavily sedated requesting a specific hospital to avoid meeting anyone they knew.

Participants’ busy and inflexible timetables were perceived as practical barriers to help-seeking, making it difficult for them to make appointments during the day as they prioritised their studies over their mental health. When students finally sought help, this did often result in treatment, either the commencement of pharmacotherapy and/or counselling. Unfortunately, however, even when students’ symptoms were identified and they were known to services, professional support in terms of counselling could be delayed due to long waiting times. In the following student’s case, this delay resulted in a deterioration in symptoms with serious consequences for their quality of life and studies:

*And then they just sort of got me in contact with the university counselling service alongside my GP, so I went to my GP who referred me all the way back to my university counselling service. The wait time was quite long, so I think it was a case of waiting for that, and then just while this was happening I think were just getting worse and worse to the point where during my second year I just pretty much didn’t care, stopped attending lectures, and yeah just sort of stopped doing work, I just found it difficult to leave bed. (Participant 5)*

#### Fear of damage to career

A minority of students felt their medical school had a caring environment, where promotion of available mental health services was built into the curriculum. For example, some students described how their medical school provided reassurance that seeking support for mental health would not negatively affect their progression towards becoming a doctor. However, most students were apprehensive about the consequences of seeking help for a mental health issue. Students feared receiving a mental health diagnosis because of its permanency and potential harm to their future career:

*I just didn’t want that label. I didn’t want to be a medical student who had a condition, it just felt really like ‘oh I don’t want to have that on there, and I just kept worrying about … because as I got to third and fourth year I knew that I really liked things like GP and psychiatry so I kept thinking well I don’t think anyone would want a GP or psychiatrist who’s got this thing going on that they call a disability or that needs medication, so it was kind of a problem of accepting it in that kind of way. (Participant 2)*

Students believed it would harm their career for three reasons. Firstly, as illustrated above, there was a belief that their patients would not want to be treated by a doctor with a mental health condition. Secondly, there was an expectation of negative judgement and treatment by the medical school. Thirdly, students feared having to face a Fitness to Practise panel. [In the UK, Fitness to Practise is regulated by the General Medical Council (GMC). An overview of relevant GMC guidance in relation to medical students can be found in the following documents: Professional behaviour and fitness to practise: guidance for medical schools and their students [15] and Supporting medical students with mental health conditions [16]].

As a result of these fears, several students chose not to approach their medical school for support for fear of repercussions related to their mental health, despite the fact that these fears were generally unfounded. Students expressed a belief that information would follow them for the remainder of their time at medical school. Sometimes this lack of trust in the medical school was generated from personal experience, but often it was overhearing experiences from peers:

*I know a friend of mine in first year was really struggling … there was nothing put into trying to help her manage and get back in and carry on with the course, it was just straight away like – just take some time out and then come back if you can, and it was very depersonalised. When you hear about that that puts you off from talking about it because you think I’ve worked so hard to get here I don’t want to lose my place, I’ll just keep quiet about it. (Participant 17)*

The fear of the medical school finding out information about a student’s mental health issues could lead to apprehension about accessing other support services, such as University Counselling services because of uncertainty about the confidentiality of their information and concern it may ‘get back’ to their medical school.

Fear of Fitness to Practise was paramount. It attracted a stigma because it calls into question whether a student is well enough to practise. Also, because Fitness to Practise proceedings can be called for a variety of reasons, including criminal behaviours, physical or mental health conditions are given the *‘moral equivalence of like committing a crime’ (Participant 7)*. Moreover, when a student’s mental health issue has led to a fitness to practise case this would lead to uncertainty about being able to practise as a doctor in the future:

The biggest problem is I still don’t know whether I’m going to be able to work in August, because the General Medical Council, they have to decide that after my exams.

(Participant 7)

### Theme 3. Impact on academic life

#### Effect on studies

With the well-recognised issues of workload and time constraints within undergraduate curricula [17], it is unsurprising that students reported falling behind as *‘the worst thing about having mental illness in medical school’ (Participant 8)*. Inability to keep up with their studies could include not being able to study outside of timetabled teaching, or not even being able to attend university at all:

And then by the first year of university I wasn’t even able to come in anymore, I think it was about two or three weeks before my first-year exams that I just went home and I just wasn’t able to do anything – I wasn’t sleeping or eating, I couldn’t study or anything like that.

(Participant 2)

Students reported cognitive challenges such as struggling with memory, attention, and difficulties concentrating which had a negative impact on their learning. The physical implications of some mental health issues could also contribute to problems with studying, for example feeling physically ill due to an eating disorder.

Participants also described their mental health issues being a contributory factor to not passing exams. Even attending exams could be difficult due to being unwell at the time:

‘And yeah I remember for those like Christmas exams as well, like genuinely just like considering just not turning up to them because I was just at this point I was just having panic attacks all the time’.

(Participant 11)

There were also implications for students’ future career plans. When feeling unwell it was difficult to fulfil the expectations of undertaking extra-curricular activities which are perceived to be important for their future medical career. Ultimately, falling behind on studies because of mental health issues can add to the already negative feelings that an unwell student may have about themselves, creating a vicious circle of negative thoughts and outcomes. The expectations students reported of the need to undertake extra-curricular activities, and concerns about being unable to fulfil these when unwell, is a further illustration that students perceive a great burden and pressure to perform in all aspects of their life, at all times.

#### Time out from medical school

Most participants talked about time away from medical school, either as something that they or someone they knew had experienced. In several instances, the option of time away was suggested by the medical school, particularly in the case of more moderate or severe mental health issues:

I think it got to a point where the medical school had offered to … well not offered but essentially recommended that I defer and don’t sit my exams because it was quite clear that I was probably going to fail.

(Participant 5)

One participant initially resisted taking time out but was so unwell they eventually had to; in retrospect they felt that that was the best thing that could have happened to them at that time, allowing access to specialist treatment. Many participants felt, however, that time away was not the best option for them. Not choosing to take time out when it was suggested could leave students feeling unsupported if this was the only real option that the medical school could provide. For example, one student explained their mental health issue is triggered by a recurring event, and so time away would not necessarily help if the triggering event cannot be changed. Taking time out generally meant re-sitting a year, which incurs significant extra expenses such as an additional year’s tuition fees and living costs. Re-sitting a year also has implications for student confidentiality, as the student can be fearful of having to disclose to others the reasons for taking time out:

‘I kind of saw it as if I have this problem I’ll end up having to retake the year of medicine and everyone will know that I’ve retaken the year, and then I might have to tell them why and what’s happened and things. So I just really didn’t want that to be an option for me’.

(Participant 2)

## Discussion

### Statement of findings

This is an original study, using in-depth qualitative interviews of medical students with mental health problems to understand the impact of mental ill-health on their experience and training at medical school. The combination of intense work demands, competitiveness, stigma and a culture where students feel they must ‘suck up’ the challenges because that is what is expected in a medical career, triggered or exacerbated existing vulnerabilities to mental ill-health. Medical culture impacted help-seeking attitudes and behaviours because of the stigma associated with mental ill-health in the medical profession, fear of Fitness to Practise procedures and potential damage to students’ medical careers. As a result, students actively hid their symptoms from the medical school and often their peers and tried to cope alone. Sadly, this frequently meant help-seeking was delayed and symptoms escalated in severity. This had a considerable influence on medical students’ academic life. Symptoms negatively affected learning which could result in falling behind in their studies and failing exams. For some, mental health issues interrupted their ability to progress through medical school.

### Comparison with existing literature and implications for practice

The hidden curriculum is an important part of medical culture, and refers to the ‘processes, pressures and constraints which fall outside … the formal curriculum, and which are often unarticulated or unexplored’ [18]. A hierarchical and competitive culture has been identified previously as a feature of the hidden curriculum at medical school [19]. Some students in our sample identified the grading system, which involves being ranked compared to their peers, contributed to the competitiveness and their feelings of inadequacy. Changing the grading system from graded (A,B,C,D,E,F) to pass/fail, has been shown to result in improved psychological wellbeing and improved satisfaction with medical education [20]. A study of seven U.S. medical schools found students in schools using grading scales with three or more categories, reported greater stress, were more likely to have burnout and to have seriously considered leaving medical school in comparison with those in schools using a pass/fail system [21]. This suggests medical schools who are using hierarchical grading could consider the use of pass/fail grading.

For many students in our sample, symptoms worsened over time and the impact became more severe. Our findings indicate the need for early intervention to address mental well-being integrated into the curriculum. One strategy could be the use of reflective practise group sessions such as Balint groups or Schwartz rounds where students can hear the experiences of others, including their peers and seniors [22]. A national evaluation of Schwartz rounds in UK healthcare staff found a statistically significant improvement in well-being [23]. Promising results from changing the curriculum and learning environment have also been reported. Slavin et al. [24], discuss their intervention to improve well-being in medical students at Saint Louis University School of Medicine, which focused on three areas: 1) Reducing stressors and improving the learning environment; 2) Equipping students to better cope with stress and provide psychological resources; and 3) Creating opportunities for students to find meaning in their work. Initiatives included changing the grading system to a pass/fail, reducing curriculum hours by 10% and introducing electives and learning communities. First year students undertook a mindfulness and resilience curriculum which was revisited at key transition points. There was also a focus on reducing cognitive distortions such as perfectionism, and students were encouraged to focus on the learning and meaning of their work, rather than results. Data was collected from students over a 10-year period. Rates of depression and anxiety in first year medical students reduced by 85% and 75% respectively. There was also an improvement in academic performance as measured by exam scores.

It is estimated that despite one-quarter of medical students having depression or depressive symptoms, only around one-sixth seek treatment (psychotherapy or pharmacotherapy) [3,25]. A reluctance to seek help extends into a doctor’s post-graduate career, with junior doctors being unwilling to seek help until they are ‘on the edge’ [26]. Addressing barriers to help-seeking is of paramount importance and reducing stigma towards mental ill-health and addressing misconceptions about the consequences of diagnoses on medical careers is key to medical students being able to speak openly and feel able to seek help. There is some evidence to suggest that medical schools increased communications about mental health during the COVID-19 pandemic and that this in turn reduced stigma, as well as increasing knowledge about support services, suggesting an increased focus on mental health communication and awareness would be beneficial [12]. Another mechanism to reduce stigma is for senior doctors to openly disclose and share their experiences of mental ill-health. Medical students exposed to physicians who self-disclose a history of mental ill-health can lessen stigma towards mental illness and lead to more positive attitudes towards help-seeking [27,28].

Medical students with mental ill-health also identified practical barriers to help-seeking. While medical schools did promote self-care and well-being initiatives, particularly during the COVID-19 pandemic [12], because of their busy schedule, students did not have the time to look after themselves, and the lack of flexibility in their timetable meant taking time to schedule appointments was challenging. Creating space in the timetable for self-care and to seek treatment, and provision of counselling services out of hours so students can attend more easily would reduce the barriers to help-seeking.

This study found that symptoms of mental ill-health are often exacerbated because of the pressure of exams. It would therefore seem particularly important to provide extra support to students with mental health issues at exam time. Thiemann et al. [29] investigated the effect of proximity on final year exams and its influence on depression and anxiety. Medical students whose final exams were imminent had significantly higher prevalence of depression and anxiety, compared to those who had exams longer than two months away, or had already been taken. This suggests that for some students the symptoms of mental ill-health may to some extent be transitory and may benefit from targeted support and open discussion with medical educators about challenging times, such as exams and death of a first patient [29,30].

Another important finding in our study was the delay to progression through medical school because of mental ill-health, with some students having taken a year out. Carr et al. [31] interviewed medical students with experience of academic interruption and found that mental health was a significant contributing factor. Our findings showed that for some of the students, a leave of absence can be beneficial. Five of the students had taken time out and thus our findings are tentative. Several students raised concerns about being asked to take time out by their medical school. While time out can be beneficial, and for some students in the acute phases of their illness be a necessary intervention, for others it may inadvertently exacerbate symptoms due to the removal of social support and lack of opportunity for work engagement [32]. It also has serious financial implications and is therefore only feasible if the student or their family has the financial resources to be able to afford it, as it means an additional year’s tuition fees and living costs. Further, it arguably reinforces stigma by indicating that someone with a mental health issue is not able to engage in medical training, thus adding to the deterrents from seeking help [25]. Due to the possible negative consequences of a period of interruption, it’s important medical schools focus on providing reasonable adjustments such as programme modifications to allow students to continue to engage with their studies [25].

### Strengths, limitations, and suggestions for future research

In terms of strengths, firstly, the study is original, as studies to date have not focussed specifically on the lived experience of medical students with mental health issues. Secondly, participants were purposefully sampled and were diverse in terms of their mental health issue, location of medical school and demographics. This diversity is considered a strength as prior research into medical students predominantly focuses on burnout, depression and anxiety with little attention paid to students with other mental health conditions. However, the diversity does introduce heterogeneity into the sample which can be considered a limitation of the study. Whilst the sample is heterogeneous in terms of the diversity of mental health issues, they are homogenous in terms of all self-identifying with a mental health issue and being a medical student at a UK medical school. Further, none of our participants reported only experiencing one mental health issue, they all reported multiple mental health issues, meaning it would have been challenging to only select those reporting one mental health issue and this sample would not have been representative of the students volunteering for the study. In addition, the reflexive thematic analysis shows that regardless of the type of mental health issues, students experiences were similar. Lastly, we took several steps to maximise the trustworthiness and rigour of the study following the four strategies outlined by Lincoln and Guba [14] of credibility, dependability, transferability and confirmability. In terms of *credibility*, this included interviewer credibility (interviews were experienced in interviewing and analysis), prolonged engagement (interviews were organised to build trust and rapport with each participant from initial email contact to arrange the interview and by using soft introductory questions), and external check (a steering group met regularly throughout the project with wide-ranging expertise including representation from medical students). Regarding *dependability*, this included rich description of study methods (for further detail please see [12]). For *transferability*, we hope to have provided a thick description by providing our findings in detail, supported by quotations to allow the reader to judge whether they feel the results could be transferred to other contexts or settings. In terms of *confirmability*, this included steps to maximise reflexivity (regular peer debriefing sessions were held during the stages of data collection and analysis to discuss our interpretations and provided an opportunity for reflection), audit trail (we kept detailed notes during data collection and analysis with rationale for key decisions such as development of concepts and themes), and triangulation (researchers have diverse backgrounds in order to reduce bias and draw on multiple perspectives).

Regarding limitations, due to ethical concerns, students were advised against participating if they were acutely unwell and thus the participants in this study may have been coping better than others. In addition, due to our purposeful sampling method where we prioritised seeking a sample with self-reported diverse mental health issues, the sample is not necessarily representative of the prevalence of mental health disorders in medical schools.Our study was an in-depth exploration of 20 medical students in eight UK medical schools. The concept of data saturation has typically been a criterion in qualitative research for ceasing data collection and/or analysis. However, Braun and Clarke [33] have recently outlined that for reflexive thematic analysis, data saturation is not considered a useful or a theoretically coherent concept and recommend adopting the concept of information power [34]. Malterud et al.. (2016) propose that the larger information power the sample holds, the lower the number of participants needed, and vice versa. Following the principles of information power, we consider the number of students interviewed to be sufficient given the narrow study aim, the purposeful sampling strategy which meant the sample selected had characteristics highly specific to the study aim, use of experienced interviewers familiar with the topic area and the use of reflexive thematic analysis to conduct an in-depth analysis.

Future research could comprise a larger scale study, perhaps including an international comparison of medical schools to examine whether similar findings emerge.

## Conclusion

Our findings showed that medical students with mental health issues felt the culture of medicine contributed to their distress and made them reluctant to seek-help, leading to a worsening of their condition. There was a negative impact on their medical education, as many struggled with their degree, and some were required to take a leave of absence. Given the high prevalence of mental health issues within medical students, addressing the factors at medical school that contribute to this distress and the barriers to help-seeking should be a priority.

## Supplementary Material

Supplemental Material

## Data Availability

Excerpts of the transcripts relevant to the study have been made available within the paper. Participants were informed that access to audio recordings and transcriptions was limited to the research team and thus are not able to be shared.
