# Supplementary material for: The experiences of students with mental health difficulties at medical school: a qualitative interview study
Source: Med Educ Online. 2024 Jun 13;29(1):2366557. doi: 10.1080/10872981.2024.2366557 (PMC11177711; doi:10.1080/10872981.2024.2366557)
Supplement: Supplemental Material [file ZMEO_A_2366557_SM7786.zip › Supplementary files/COREQ.docx]

**Table 1**

Consolidated criteria for reporting qualitative studies (COREQ): 32-item checklist

| **No** | **Item** | **Guide questions/description** |
| --- | --- | --- |
| **Domain 1: Research team and reflexivity** |  |  |
| Personal Characteristics |  |  |
| 1. | Interviewer/facilitator | AR, RV, MS |
| 2. | Credentials | AR – Doctor of Psychology (Health), RV - PhD, AM - PhD, MS – PhD, AG – Doctor of Education & medical doctor (MBBS) |
| 3. | Occupation | AR – Associate Professor, RV – Associate Professor, AM – Senior Research Fellow, MS -Assistant Professor, AG - Professor |
| 4. | Gender | All female |
| 5. | Experience and training | AR, RV and MS had previous experience of interviewing for qualitative research and conducting reflexive thematic analysis. |
| Relationship with participants |  |  |
| 6. | Relationship established | No |
| 7. | Participant knowledge of the interviewer | Participants knew that all researchers worked for UCL, their job titles and their role within the study. Participants all received an information sheet about the study. |
| 8. | Interviewer characteristics | Interviewers’ professional backgrounds are provided in the paper. |
| **Domain 2: study design** |  |  |
| Theoretical framework |  |  |
| 9. | Methodological orientation and Theory | Reflexive thematic analysis |
| Participant selection |  |  |
| 10. | Sampling | Purposive |
| 11. | Method of approach | Participants completed a questionnaire and then were sent an email |
| 12. | Sample size | 20 |
| 13. | Non-participation | None of the participants dropped out or refused, although some did not respond to the initial email inviting participation in the study. |
| Setting |  |  |
| 14. | Setting of data collection | Microsoft Teams |
| 15. | Presence of non-participants | None |
| 16. | Description of sample | Demographics are provided in Table 1 in the paper. |
| Data collection |  |  |
| 17. | Interview guide | The research team developed the interview guide with input from the Steering Group which comprised two medical student representatives, in addition to members from Medical Schools Council, Practitioner Health Programme, British Medical Association and academics with experience of researching medical student mental health. |
| 18. | Repeat interviews | None |
| 19. | Audio/visual recording | Audio and video recordings using Microsoft Teams. |
| 20. | Field notes | Yes notes were made during the interviews. |
| 21. | Duration | Interview duration ranged from 35 to 61 minutes (mean = 50 minutes). |
| 22. | Data saturation | No – we drew on concept of information power as described in the paper. |
| 23. | Transcripts returned | No |
| **Domain 3: analysis and findings** |  |  |
| Data analysis |  |  |
| 24. | Number of data coders | 3 |
| 25. | Description of the coding tree | Authors developed a coding framework as is described in the ‘analysis’ section of the methods. |
| 26. | Derivation of themes | Themes were derived inductively. |
| 27. | Software | NVivo 12. |
| 28. | Participant checking | No |
| Reporting |  |  |
| 29. | Quotations presented | Quotations are presented in results to illustrate the themes |
| 30. | Data and findings consistent | Yes, we present the themes with supporting quotes to illustrate our findings. |
| 31. | Clarity of major themes | Yes – please see results |
| 32. | Clarity of minor themes | Yes – sub-themes are presented in the results |
