# Supplementary material for: The experiences of students with mental health difficulties at medical school: a qualitative interview study
Source: Med Educ Online. 2024 Jun 13;29(1):2366557. doi: 10.1080/10872981.2024.2366557 (PMC11177711; doi:10.1080/10872981.2024.2366557)
Supplement: Supplemental Material [file ZMEO_A_2366557_SM7786.zip › Supplementary files/Interview_guide.pdf]

Interview schedule front page

Interviews with medical students who have experienced/are experiencing mental health issues

Aims of study

In this study we are aiming to identify the types and prevalence of mental health issues experienced by medical students, and to identify factors that can provoke or prevent these issues. We are doing this using questionnaires over a year-long period, analysing university policy documents, and by speaking to medical students who have experienced mental health issues during their studies.

This research will identify the things that are associated with students' mental health and wellbeing at medical school, with the resulting aim of developing recommendations about promoting a culture that will enhance wellbeing.

To that end we would like to hear about your experiences of mental health and wellbeing, and about your experiences of medical school in relation to this.

Since this project was originally conceived, the Covid-19 pandemic took hold and changed lots of things about our home, work, and education lives. We are aware that there may be issues that have arisen out of this, and so we are interested to hear about anything related to this as well.

Yours and others' anonymised views and experiences, together with the results from the questionnaire and university policy documentation, will be written up in a report for the British Medical Association, and some findings may be published in academic journals and presented at conferences for the wider medical school community to learn from and develop improvements.

Process

Over the next hour or so, I will ask you some questions about your experiences which will hopefully spark some interesting conversations. Before we get started, I want to highlight a few things:

- You can turn the video off at any time during the interview if you would prefer to talk without it
- If you see me look away, I will be looking at the interview schedule to check that I'm asking the questions correctly
- There are no right or wrong answers, I just want to hear your opinions and experiences.
- I'd like to audio record our conversation and take some notes, to help me accurately remember what was said.
- What you say will be kept confidential – we won't share details of this interview with anyone outside of the research team.
- The recording of your interview will be transcribed and anonymised; only the audio will be sent to the transcriber, they will not receive the video and the anonymised transcripts will be analysed by the research team in the Research Department of Medical Education at UCL.
- We may publish small amounts of what you say in our research report and papers, but this will only be done in a way that makes it impossible for anybody to identify you as the person who said it.

CONFIDENTIAL

- If you want to stop or you need to leave at any time during the interview, that's absolutely fine.
- If during the interview we talk about something that leads me to worry about immediate harm to yourself or to others, we will stop the interview and talk about finding support for this instead.

Do you have any questions about any of that?

Can I check that you have read the participant information sheet and completed the consent form?

Can I check that you are not acutely unwell at the moment? (If Y, suggest postponing interview)

Before we start, I would like to check that the details we have for you are correct. Check participants:

You said you have experienced the following:

Depression / Anxiety / Insomnia / Stress / Burnout / Eating Disorder / Drug or Alcohol use / Personality disorder / Bipolar / Other

Confirm the following demographics:

University / Age / Gender / Ethnicity / Sexuality

I'm now going to turn the recording on [TURN ON RECORDER]

Topic guide

The first four questions are to help warm-up, and provide some context, we do not need to cover all of them (e.g. can omit the specialty question) if the participant is already warmed up by the first questions.

Why did you decide to study medicine?

Why did you choose this university?

How have you found medical school so far?

Are you interested in any particular specialties yet?

The following questions form the main part of the interview.

Can you tell me about your experiences of mental health and wellbeing?

Prompts:

- *They don't have to have just been at university, we're interested to hear about them if they began before coming to medical school as well.*

CONFIDENTIAL

Can you tell me about your experiences of mental health and wellbeing while at medical school?

Prompts:

- Was there something that triggered or exacerbated things while at medical school?
- Is there a positive experience you'd like to talk about?
- Is there a negative experience you'd like to talk about?

Were you able to talk about your [experiences/feelings] with anyone?

Prompts:

- Who, and why?
  - *Push on* ***why not other people*** *– e.g. if talk with friends/family, why not tutors/student support. If student support/tutors, why not friends/family.*
- If not, why?

NB: if anyone mentions/describes ***<u>stigmatisation</u>*** *of mental health issues, unpack:*

- E.g. Why do you think [it's stigmatised]?; what negative impact do you think this status of 'mentally ill' has?; do you have any direct examples, or something that felt within?

Did you seek any kind of formal support for your [experiences/feelings]?

Prompts:

- Could you just talk me through your decision a bit?
- *If yes:* What influenced you to seek formal support? E.g. People/media?
- *If no:* Why didn't you seek formal support?

What was your experience of support?

Prompts:

- Within the medical school?
- Within the university?
- External sources?
- Family and friends?
- Was there anything that you found especially helpful at medical school?
- Was there anything that you found especially unhelpful at medical school?

How would you describe the culture or attitudes at medical school towards students' mental health and wellbeing?

Prompts:

- How about towards students experiencing mental health issues?

Is there anything that you think medical schools can do to better support students when experiencing mental health issues?

CONFIDENTIAL

Is there anything that you think medical schools can do to protect students' mental health/prevent them from developing mental health issues?

CONFIDENTIAL
